# Biochemical Characteristics of New Delhi Metallo-β-Lactamase-1 Show Unexpected Difference to Other MBLs

**DOI:** 10.1371/journal.pone.0061914

**Published:** 2013-04-12

**Authors:** Tao Li, Qin Wang, Fanghong Chen, Xiang Li, Sen Luo, Huali Fang, Dehui Wang, Zhan Li, Xiaojun Hou, Hui Wang

**Affiliations:** State Key Laboratory of Pathogens and Biosecurity, Beijing Institute of Microbiology and Epidemiology, Fengtai District, Beijing, People's Republic of China; Aligarh Muslim University, India

## Abstract

New Delhi metallo-β-lactamase (NDM-1) is a new metallo-β-lactamase (MBL) that has recently emerged as a global threat because it confers bacteria with resistance to almost all clinically used β-lactam antibiotics. To determine the molecular basis of this threat, NDM-1 was purified from *Escherichia coli* TransB (DE3) carrying cloned blaNDM-1 gene by an anion-exchange chromatography step followed by a gel permeation chromatography step. The purified enzyme was stable even in extremely alkaline buffer (pH 11) and reached its highest activity at a low temperature (15°C), which was different from other MBLs. The 50% inhibition concentration of EDTA against NDM-1 was 412 nM, which showed that NDM-1 was more susceptible to EDTA than other MBLs. The effects of zinc on NDM-1 differed between cephem and carbapenem complexes, but inhibition at high Zn^2+^ concentration was observed for all of tested β-lactam compounds.

## Introduction

New Delhi metallo-β-lactamase (NDM-1) was first reported in a Swedish patient in 2009. This patient traveled to New Delhi and acquired a urinary tract infection caused by *Klebsiella pneumoniae* [1]. Cases of the spread and dissemination of NDM-1-positive *K. pneumoniae* strains have been reported worldwide since August 2010 [2]. A recent study has reported the high resistance of NDM-1-positive *K. pneumoniae* and *Escherichia coli* strains to all tested antibiotics, except tigecycline and colistin [3]. Therefore, the spread of pathogenic microorganisms carrying the NDM-1 gene (also called "super bugs") is a major global health threat.

NDM-1 belongs to the metallo-β-lactamase (MBL; class B) family, which contains Zn^2+^ and other divalent cations as cofactors. The strain inactivates almost all classes of β-lactam antibiotics, including carbapenems, by catalyzing the hydrolytic cleavage of the substrate amide bond. Several experimental and theoretical studies aimed at understanding the properties of MBLs have been conducted [4]–[7]. NDM-1 is more potent in inactivating β-lactam antibiotics than known MBLs. Thus, to develop antibiotics that can combat emerging NDM-1-positive pathogens, the catalytic mechanism of NDM-1 needs to be determined.

In this study, NDM-1 was purified and characterized to explore its molecular basis for antibiotic hydrolysis. A system of NDM-1 overproduction in *E. coli* and a simplified purification protocol were developed. The enzymatic activity of NDM-1 was determined based on a large number of substrates. The effects of EDTA and Zn^2+^ on the enzyme activity, as well as the pH and temperature dependence of the zinc content, were also measured.

## Materials and Methods

### Plasmids, strains, and reagents

*E. coli* TransB (DE3) chemically competent cells, *E. coli* trans5α chemically competent cells, and pEASY-T1-simple cloning vector were purchased from Beijing TransGen Biotech (China). Taq DNA polymerase, T4 DNA ligase, and restriction endonucleases were obtained from New England Biolabs (Beijing, China). PCR primers were synthesized by Beijing Sunbio Tech Co., Ltd. (China). Plasmid mini-kits and gel extraction kits were obtained from Beijing Biomed Co., Ltd. (China). Q-Sepharose FF and Superdex 75 columns (5 ml) were purchased from GE Healthcare (Beijing, China). The expression vector pET22b was stored in our laboratory. All other chemicals and reagents were obtained from commercial sources and were of the highest purity available.

### Recombinant DNA methodology

ATCC BAA-2146 was used as the bacterial strain for the PCR amplification of the *ndm-1* gene. PCR was performed using the primers P1 (5′-CATATGAAGTGTATATTATTTAAATGGG-3′) and P2 (5′-CTCGAGTCAGCGCAGCTTGTCGGC-3′). Amplification of the *ndm-1* gene by PCR with P1 and P2 was performed using EasyTag polymerase under the following conditions: initial denaturation at 95°C for 3 min; followed by 30 cycles at 94°C for 30 s, 60°C for 30 min, and 72°C for 1 min; and final renaturation at 72 °C for 5 min. The PCR product was subcloned into the pEASY-T1 cloning vector. *ndm-1* gene in the recombinant plasmid pEASY-*ndm1* was cloned into the expression vector pET22b as an *NdeI*–*XhoI* fragment. The sequence of the plasmid pET22b-*ndm1* was confirmed by DNA sequencing. The expression of plasmid pET22b-*ndm1* was screened in DH5α and then expressed in *E. coli* TransB (DE3).

### Expression and purification of NDM-1 protein

*E. coli* TransB (DE3) harboring the expression plasmid pET22b-*ndm1* was grown at 37°C in one liter of Luria–Bertani medium containing 100 µg/ml ampicillin with shaking until the OD_600_ reached 0.4–0.6. Protein expression was induced by adding IPTG to a final concentration of 0.1 mM for 8 h at 25°C with shaking. Cells were harvested by centrifugation, and the supernatant was removed by decantation. The pellet was resuspended in 250 ml of buffer A (20 mM Tris-HCl, pH 7.4), and mild sonication was conducted on ice. After centrifuging the lysate, the supernatant fraction and pellet fraction were resolved by SDS-PAGE using a 15% acrylamide gel.

The supernatant fraction containing the required proteins was loaded onto a Q-Sepharose FF column on an ÄKTA Explorer™ station (GE Healthcare, USA). After washing the column, the bound proteins were eluted by a linear NaCl gradient (0 M to 1 M) in Tris-HCl buffer (flow rate = 1 ml/min). The fractions containing carbapenemase activity were pooled, dialyzed against 10 mM HEPES buffer (pH 7.5), concentrated 10-fold by ultrafiltration, loaded on an equilibrated Superdex 75 column, and eluted with 10 mM HEPES (pH 7.5). The β-lactamase-containing elution peak was analyzed by SDS-PAGE (15% acrylamide gel) and Bandscan 5.0 software. The protein concentration in solution was assayed with a commercial kit (Bio-Rad protein assay). During the purification procedure, carbapenemase activity was monitored using 100 mM meropenem as a substrate in 50 mM HEPES (pH 7.5) at 30°C.

### Kinetic measurements

Purified NDM-1 was used to determine the kinetic parameters *K*_cat_ and *K*_m_. Reactions were performed at 30°C in 0.2 ml of assay buffer (pH 7.5 50 mM HEPES and 50 µM ZnSO_4_). The assays were performed using a SynergyHT spectrophotometer (Biotek Corp., USA) by observing changes in absorption resulting from the opening of the β-lactam ring at specific wavelengths for each antimicrobial agent evaluated. The observations were made every 1 min for 10 min as previously described [8]. The results are presented as the average±standard deviation based on three independent measurements.

### Temperature and pH effects

To determine the effects of temperature on enzyme activity, 50 µg/ml purified NDM-1 was incubated for 20 min before assaying with 0.5 mM meropenem in a buffer (pH 7.5 50 mM HEPES and 50 µM ZnSO_4_) from 5°C to 70°C. To determine the effects of pH on enzyme activity, purified NDM-1 was incubated in 50 mM HEPES buffer from pH 5.5 to 11 for 20 min before assaying with 0.5 mM meropenem at 30°C under the aforementioned conditions. Averages and standard deviations were reported based on three independent measurements.

### IC_50_ determination using EDTA

To examine the effects of chelation by EDTA, an enzyme assay was performed by determining the IC_50_, which is defined as the concentration of inhibitor necessary to inhibit 50% enzyme activity. The assay was performed under previously described conditions after incubating the reaction mixture for 10 min with varied concentrations of EDTA from 0.1 µM to 1 µM before initiating the reaction with meropenem.

### Effects of zinc concentration on various substrates

The initial hydrolysis rates of each substrate were determined by adding NDM-1 protein to an assay buffer containing varied amounts of ZnSO_4_ from 0.2 µM to 200 µM. The final enzyme concentration was 0.5 µg/ml. Averages and standard deviations were reported based on three independent measurements.

## Results

### NDM-1 expression and purification

**Figure 1:**
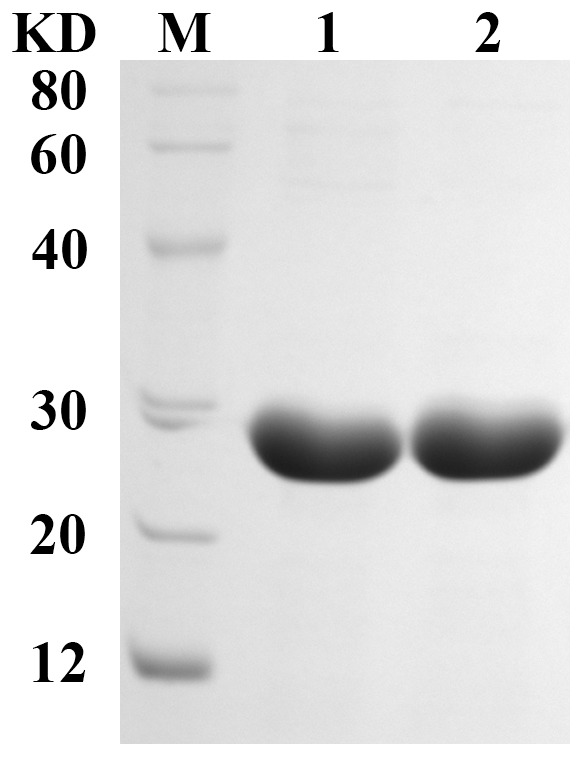
Coomassie blue stained 12% SDS-PAGE of purified NDM-1. The highly purified and soluble NDM-1 is shown. The molecular weight of NDM-1 is approximately 28 kDa.

NDM-1 enzyme was purified from a lysate of *E. coli* TransB (DE3), which carries cloned blaNDM-1 gene on the expression plasmid vector pET22b, by an anion-exchange chromatography step on Q-Sepharose. Then, a gel permeation chromatography step on Superdex 75 was performed. Approximately 95 mg of purified enzyme was obtained per liter of culture using the above-described protocol. The degree of purity, as evaluated by SDS-PAGE, was >95% (Fig. 1); the overall yield of the purification protocol was 17%. In SDS-PAGE, NDM-1 polypeptide migrated with an apparent molecular mass of approximately 28 kDa (Fig. 1).

### Kinetic parameters of NDM-1 with various β-lactam compounds

The kinetic parameters of NDM-1, including *K*_m_, *k*_cat_, and the *k*_cat_/*K*_m_ ratio, were determined using different β-lactam compounds. Under the adopted experimental conditions, the enzyme hydrolyzed all tested compounds (Table 1). The observed NDM-1 catalytic efficiencies with cephem and carbapenem were 0.6–0.8 µM^−1^ s^−1^, and those obtained with penam were lower (0.1–0.3 µM^−1^ s^−1^). The *K*_m_ values of NDM-1 were similar among different β-lactams, but the *k*_cat_ values differed by up to 12-fold. The highest *k*_cat_ (105.6 s^−1^) was found with biapenem and the lowest (8.3 s^−1^) was with piperacillin. Considerable variability was observed within the various β-lactam subfamilies (penam, cephem, and carbapenem compounds). Thus, NDM-1 had no clear preference for any of these β-lactam compounds (Table 1).

**Table 1:** Steady-State Kinetic Constants for NDM-1 and Various Substrates

substrate class	substrate	nm	*K_m_*( µM)	*k_cat_*(s^−1^)	*k_cat_*/*K_m_*( µM^−1^s^−1^)
penem	penicillin G	235	103	27.5	0.27
	piperacillin	230	69	8.3	0.12
cephem	cefoxitin	230	63	38.3	0.61
	ceftazidime	260	74	60.9	0.83
carbapenem	cefotaxime	260	43	31.5	0.74
	biapenem	294	155	105.6	0.68
	meropenem	297	42	30.75	0.73

### Effects of temperature and pH on enzyme activity and stability

**Figure 2:**
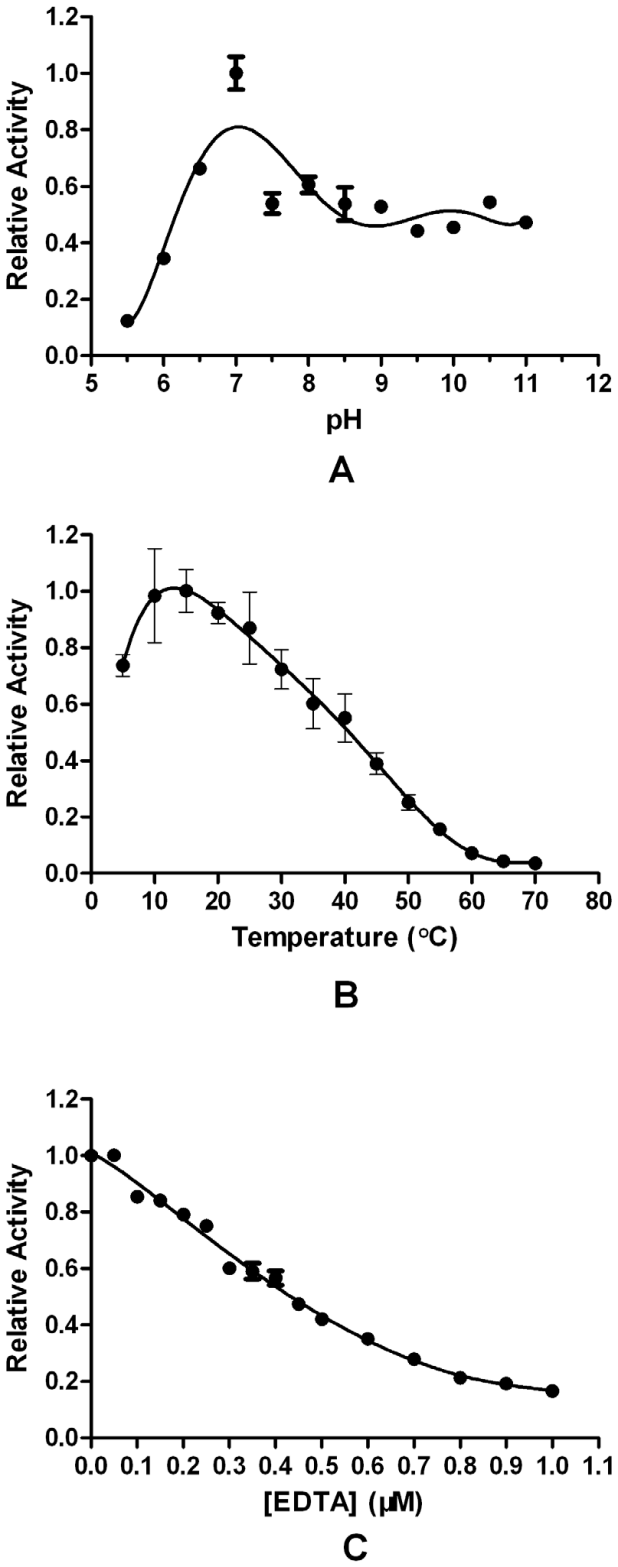
Effects of the pH, temperature, and EDTA on NDM-1. A: pH dependence of NDM-1 at 25°C. The enzyme was incubated in buffers with various pH values for 20 min at 30°C before assaying with 0.5 mM meropenem as a substrate. Averages are reported based on three independent measurements. B: Temperature dependence of NDM-1 at pH 7.5. The enzyme was incubated at different temperatures (5°C to 70°C) for 20 min before assaying with 0.5 mM meropenem as a substrate. Averages are reported based on three independent measurements. C: Determination of the IC_50_ of EDTA against NDM-1. The enzyme (0.5 mg/ml NDM-1) was incubated in buffers with various EDTA concentrations [final = 10 mM HEPES (pH 7.5)] for 10 min at 30°C before assaying with 0.5 mM meropenem as a substrate. The IC_50_ value is approximately 412 nM.

NDM-1 had the highest activity between pH 6.5 and 8.0, with the maximum activity at pH 7.0 (Fig. 2A). Most MBLs prefer buffers with pH ranging from 6.5 and 7.0 [9]–[12]. However, NDM-1 retained >50% of its original activity at pH 11. To determine the buffer effect, NDM-1 activity in 40 mM ethanolamine buffer at pH 10 and 11 was observed. The results were similar to those in HEPES buffer. All these findings suggested that NDM-1 was stable and active at high-pH buffers. NDM-1 had the highest activity at 10 and 25°C, with the maximum activity at 15°C (Fig. 2B). Most MBLs have an optimal temperature of 37°C. However, at 35°C, NDM-1 retained only 60% of its activity at 15°C; at 55°C, less than 10% was retained. Thus, NDM-1 was unstable at high temperature.

### IC_50_ of EDTA against NDM-1

We compared the inhibition effects of EDTA on NDM-1 and on different MBLs [9], [12]. The IC_50_ of EDTA against NDM-1 was 412 nM (Fig. 2C). Therefore, NDM-1 was more susceptible to EDTA than other MBLs such as DIM-1, VIM-1, and VIM-13, whose IC_50_ values are 175, 9.3, and 253 µM, respectively. Thus, Zn ions can more strongly bind to NDM-1 than to other MBLs.

### Effects of zinc concentration on various substrates

**Figure 3:**
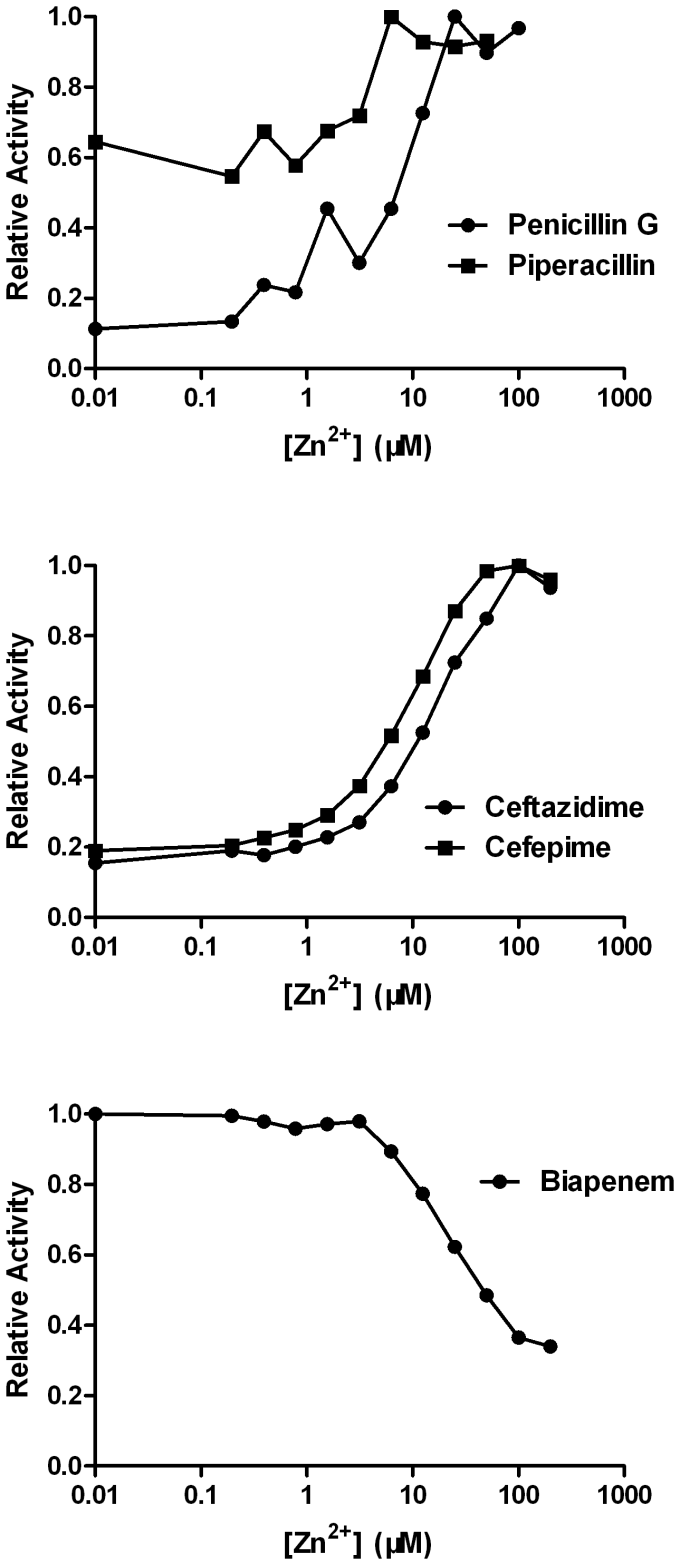
Zinc dependence of the NDM-1-catalyzed hydrolysis of various substrates. Measurements were made with 0.5 mM substrates and increasing ZnSO_4_ concentrations in the assay solution. Penems: penicillin G and piperacillin. Cephems: cefepime and ceftazidime. Carbapenem: biapenem.

To determine the effect of zinc ion stoichiometry on function, we investigated the NDM-1 ability with increased ZnSO_4_ concentration in an assay buffer used to hydrolyze three different classes of substrates (Fig. 3). The hydrolysis rates of cephem (ceftazidime and cefepime) were found to be very sensitive to the ZnSO_4_ concentration in the assay buffer. However, the hydrolysis rate was only 15%–20% of the maximum activity when no additional ZnSO_4_ was added. The rates reached the maximum level when the ZnSO_4_ concentration was 100 µM. A subset of these substrates showed increased hydrolysis rates with increased ZnSO_4_ concentration. The tested penams showed varied results. Penicillin G showed low sensitivity to the ZnSO_4_ concentration, but piperacillin showed effects similar to those of cephem. Carbapenem (biapenem) showed effects opposite to those of the other substrates, i.e., significantly decreased activity at high ZnSO_4_ concentration. When the ZnSO_4_ concentration was <4 µM in the assay buffer, the maximum activity was observed with biapenem.

## Discussion

Since the first report on NDM-1 in 2009, this enzyme has been extensively studied. However, most of these studies have focused on genetics, genomics, and molecular epidemiology. Only a few papers have been published on the functional properties of NDM-1. So far, four reports on the structural determination of NDM-1 have been published [13]–[16], with additional coordinates available in the Protein Data Bank. These structural models show that NDM-1 has a typical MBL fold, but these models considerably differ from one another. However, all these reports show that NDM-1 proteins have fusions or tags used for purification (e.g., His tag), which may affect the structure of the active site. In the current study, an engineered E. coli that carried the cloned blaNDM-1 gene on expression plasmid vector pET22b was constructed. The high yield of NDM-1 production (approximately 100 mg L−1 of culture) led to a purified NDM-1 preparation (>98% purity) that was obtained after cation exchange and gel permeation chromatography.

The pH dependence of lactamase is indispensable in elucidating the mechanism of NDM-1. Active sites generally contain acidic or basic groups, and one protonic form of the acid and base is catalytically active. The pH affects the enzyme activity through the group ion state. In this study, we found that NDM-1 was stable and active in an extremely basic environment (even pH>11.0). However, the enzyme activity in acidic buffer (pH<6.0) sharply decreased and had no significant activity at pH 5.5. Thus, appropriate acid groups should be considered in the design of NDM-1 inhibitors.

EDTA is a metallo-chelator known to potentiate the activities of some lactam antibiotics against MBL-producing microorganisms because the zinc-containing active site of MBL is inactivated by EDTA [17], [18]. In this study, we found that NDM-1 was more susceptible to EDTA than other MBLs, indicating a stronger binding of Zn^2+^ ions in NDM-1. Despite the toxicity of EDTA, which hinders its clinical use, EDTA complexes or analogs are important in finding new NDM-1 inhibitors. A complex of EDTA and calcium ion called calcium disodium EDTA has been prepared as an injectable form of chelator with decreased toxicity. This form has been approved for the treatment of lead intoxication [19], [20] and used as an inhibitor of bacterial MBL in a mouse model of *Pseudomonas aeruginosa* pneumonia [21].

The effects of zinc on NDM-1 varied between cephem and carbapenem complexes; however, inhibitory effects were observed for both complexes at high Zn^2+^ concentrations. This inhibition can be explained by the nonspecific interactions between the enzyme surface and Zn^2+^ cations, which have also been observed for VIM-2 and other MBLs [22], [23]. Low-concentration Zn^2+^ fixation strongly stabilized the enzyme and impaired the active site mobility. However, high-concentration Zn^2+^ destabilized the enzyme and disorganized the active site. Hence, the optimum Zn^2+^ concentration depended on the antibiotic used.

In conclusion, a new and simple method of purifying native (no fusion or tag) NDM-1 was demonstrated. The enzymes were successfully characterized by kinetic measurements as well as analyses on the effects of pH, temperature, Zn, and EDTA. The results showed unexpected differences of NDM-1 from other MBLs.
